# Experience with Aposematic Defense Triggers Attack Bias in a Mantid Predator (*Stagmomantis carolina*)

**DOI:** 10.1093/iob/obae039

**Published:** 2024-10-25

**Authors:** L E Schweikert, D R Chappell, Z Huang, G E Delpizzo, K Wahi, M O Saunders, V E Slye, L F Naughton, N I Rummelt, L E Bagge

**Affiliations:** Department of Biology and Marine Biology, University of North Carolina Wilmington, Wilmington, NC 28403, USA; Air Force Research Laboratory/RWTCA, Eglin Air Force Base, FL 32542, USA; National Academies of Sciences, Engineering, and Medicine, 500 Fifth St. N.W., Washington, DC 20418, USA; Department of Physics, Cornell University, Ithaca, NY 14850,USA; Lewis-Sigler Institute for Integrative Genomics, Princeton University, Princeton, NJ 08544,USA; Department of Biotechnical and Clinical Laboratory Sciences at the Jacobs School of Medicine, University of Buffalo, Buffalo, NY 14260,USA; Department of Neuroscience, Indiana University Indianapolis, Indianapolis, IN 46202, USA; Department of Biological Sciences, University of New Orleans, New Orleans, LA 70148,USA; Department of Biology and Marine Biology, University of North Carolina Wilmington, Wilmington, NC 28403, USA; Department of Biology and Marine Biology, University of North Carolina Wilmington, Wilmington, NC 28403, USA; Air Force Research Laboratory/RWTCA, Eglin Air Force Base, FL 32542, USA; Air Force Research Laboratory/RWTCA, Eglin Air Force Base, FL 32542, USA

## Abstract

Studies of predator psychology in aposematism have suggested important effects of signal detection through space and time on outcomes of attack behavior. Both the integration of aposematic signals from prey and experience state of the predator can have important effects on attack decisions. The universality of these effects however, especially as it applies to non-avian predators such as arthropods, remains poorly understood. We examined the effects of multimodal aposematic signaling and prior experience with aposematism on attack latency and attack likelihood of the Carolina mantis (*Stagmomantis carolina*). Using artificial prey bearing visual and olfactory signals of the convergent lady beetle (*Hippodamia convergens*), we tested 2 cohorts of mantids (representing juvenile and adult stages) across 4 groups: visual only, odor only, combined signals, and control. We then used approaches in linear modeling to test the hypotheses that (1) prior experience with aposematism alters attack behavior toward aposematic prey and (2) multimodal signals have synergistic effects on attack behavior relative to either unisensory signal presented alone. We found support for the first hypothesis in that mantids employ attack biases against visual and olfactory aposematic signals, but only after prior exposure to aposematism and only as juveniles. While support is lacking for multimodal integration by the mantids, this study is the first to suggest a response of mantids to an aposematic olfactory signal (in addition to visual signal) and may suggest a developmental window for mantid predators to develop biases toward aposematic prey that are shaped by experience.

## Introduction

Aposematic displays are often multimodal, wherein combined signals, across sensory modalities, may be more information rich than any one signal presented alone (Rowe and Halpin 2013). These displays are often comprised of warning colors or contrast patterns, combined with sounds, odors, or distasteful secretions solicited by attack (Cott 1940; Edmunds 1974; Ruxton et al. 2004). Aposematic signaling has been comprehensively reviewed (Rowe and Guilford 1999a; Hebets and Papaj 2005; Partan 2013; Rowe and Halpin 2013; Rubi and Stephens 2016; Winters et al. 2021), with the combination of signals predicted to enhance information content or to improve display efficacy via signal transmission and its effects upon the receiver(s) (Hebets and Papaj 2005). Receiving less attention, however, is the effects of multimodal signals on predator psychology (Guilford and Dawkins 1991): the study of how receivers use information when making attack decisions, which may have equal or greater impact on the evolution of aposematic displays than the benefits to the signalers themselves (Guilford 1992; Rowe 1999; Ruxton et al. 2004; Miller and Bee 2012; Skelhorn et al. 2016).

The predator psychology of multimodal aposematic signaling is best understood from studies of avian predation, which have given special attention to the effects of novelty and prior experience on attack decisions of aposematic prey (Rowe and Guilford 1996; Rowe and Guilford 1999b; Rowe and Skelhorn 2005). Novelty itself can elicit attack biases against those prey or to others in subsequent interactions (Schuler and Hesse 1985; Roper and Cook 1989; Marples and Roper 1996; Rowe and Guilford 1999b). This has been repeatedly demonstrated in domestic chicks (*Gallus gallus domesticus*), showing that odor novelty can induce foraging biases against warningly colored prey (Marples and Roper 1996; Rowe and Guilford 1999b; Jetz et al. 2001; Skelhorn et al. 2008). As for prior experience, attack biases can manifest either from learned association (Skelhorn et al. 2016) or the activation of a latent bias (Rowe and Guilford 1999a; Skelhorn et al. 2008). For learned association (i.e., passive avoidance learning), pairing an aversive cue with a warning signal establishes a relationship between signal and defense (Gittleman and Harvey 1980; Guilford 1990; Rowe and Skelhorn 2005). For latent biases, experiencing a chemical defense in the absence of a warning signal can elicit a non-associated or implicit aversion to a given warning signal in a later interaction (Schuler and Hesse 1985; Roper and Cook 1989; Skelhorn et al. 2008; Rowland et al. 2013). For example in domestic chicks, experiencing an olfactory or gustatory defense can result in a subsequent (and non-associated) aversion to aposematic coloration (Rowe and Guilford 1996; Rowe and Guilford 1999a; Lindström et al. 2001; Rowe and Skelhorn 2005). These studies suggest that the combination of aposematic signals has relevance over space and time (Mongeau et al. 2021) and that an evolutionary hardwiring of adaptive attack decisions may exist beyond the naïve state.

Relative to avian systems, the predator psychology of arthropods is far less understood. Studies of arthropods have focused on their role as a prey item for terrestrial fauna, with multimodal signaling in aposematism largely being studied from the perspective of the sender (see Rowe and Halpin 2013*for review*). The receiver's perspective, however, has been best studied in dragonflies (order Odonata), suggesting roles of prey size (Rashed et al. 2005; Duong et al. 2017), shape, and warning coloration (Kauppinen and Mappes 2003) in attack biases toward aposematic prey. In jumping spiders, context-dependent learning in predation behavior (Skow and Jakob 2006) and avoidance learning of warningly colored prey (Raška et al. 2017) have both been documented. Similar to unlearned aversions in the avian literature (Rowe and Guilford 1999b; Rowe and Skelhorn 2005), recent studies have shown that experiencing a novel olfactory defense in jumping spiders (*Habronattus trimaculatus*) triggers an attack aversion to warningly colored prey (Vickers and Taylor 2018, 2020). Together, these studies suggest that mechanisms in multimodal integration are shared across diverse taxa (at least in birds and arthropods; Vickers and Taylor 2020; Segovia and Pekár, 2023) and that the canonical rules of receiver psychology of aposematism may apply to both avian and non-avian predators, generally.

Praying mantids (order Mantodea) are an excellent study system for enhancing our understanding of arthropod predator psychology due to their aggressive predatory behavior and documented sensory capabilities (Sontag 1971; Rossel 1979, Prudic et al. 2007; Fabricant and Herberstein 2015), which may permit detection of multimodal signals. Mantid vision has been described as monochromatic (Prudic et al. 2007; Fabricant and Herberstein 2015), though some studies, specifically of genus *Tenodera*, have suggested two-channel spectral sensitivity in green and ultraviolet regions of the spectrum (Sontag 1971; Rossel 1979). They also have relatively high visual acuity relative to other arthropods (Land 1997; Feller et al. 2021) and an apparent chemosensory capacity, which has been implicated in sex pheromone detection (Carle et al. 2014) and prey-finding behavior (Ezaki et al. 2021). Studies of mantid predation and aposematism are limited, but at least for genus *Tenodera*, mantids have been observed attacking chemically defended prey and learning from those experiences. Specifically, attacks upon the cardenolide chemical defense of milkweed bugs (*Oncopeltus fasciatus*; Berenbaum and Miliczky 1984) yielded an emetic response in *Tenodera* mantids (Berenbaum and Miliczky 1984) and learned avoidance of contrast coloration (Bowdish and Bultman 1993; Prudic et al. 2007; Carle et al. 2018). As for multimodal integration of aposematic defenses by a mantid predator, an empirical investigation has yet to be performed.

We aimed to address the question: How do visual and olfactory warning signals interact to influence attack behavior of a mantid predator before and after a learning event? We used the Carolina mantid (*Stagmomantis carolina*) as the predator and the convergent lady beetle (*Hippodamia convergens*) as the prey due to its known visual and chemical defenses (Wheeler et al. 2015). Considering the avian literature, we hypothesized *a priori* that (1) prior experience with aposematic prey would alter attack responses of mantids to aposematic prey and (2) aposematic signals would interact synergistically so that responses to multimodal signals would differ from those to either unisensory signal. Using a repeated-measures design, we tested the attack latency of mantids to one of four signal groups, before and after being offered a live lady beetle as prey. The four groups were (1) visual only, (2) odor only, (3) combined signals, and (4) control, which were tested across two cohorts of mantids, representing juvenile and adult life-history stages.

## Materials and methods

### Study specimens

The study specimens (*N* = 47) were Carolina mantids (*S. carolina*; family Stagmomantinae) that had been captive-bred and commercially obtained from US Mantis, Inc. (New York, USA). The first cohort arrived in June 2022 and was comprised of juvenile L4/L5 nymphs (*n* = 24). The second arrived in September 2023 and was comprised of adults, one of which died upon arrival, reducing the sample size to *n* = 23. Thus, the timing of mantid acquisition is one potential confound between the cohorts worthy of consideration. During rearing, the mantids were fed flightless fruit flies (*Drosophila hydei*) *ad libitum*. Once received, juveniles were maintained on *D. hydei* and adults on blue bottle flies (*Calliphora vicina*) and, thus, were putatively naïve to aposematic prey. All mantids were fed every other day, held within a 12:12-h light–dark cycle, and tested within 14 days of arrival. Mantids experienced a normal feeding schedule prior to testing, with the experiment beginning on a scheduled feeding day in order to try to standardize their predatory motivation.

### Artificial prey

Artificial prey were casted using white modeling clay (Jovi, Barcelona, Spain) that was air-dried within a 6 × 5-mm lady beetle silicone mold (World of Sugar Art, Latham, NY, USA). To facilitate mounting, a 7 mm-long wire post was pressed into the ventral surface of the prey, before adding further modifications representing visual and olfactory warning signals. Aposematic signals relevant to the convergent lady beetle (*H. convergens*) were used for two reasons: firstly, because the visual and chemical signals of this species are honest aposematic signals (Wheeler et al. 2015) and secondly, because they are sympatric with the Carolina mantid (Lutz 1935; Sethuraman et al. 2015), providing evolutionary context for how the mantids might interact with this prey.

**Fig. 1:**
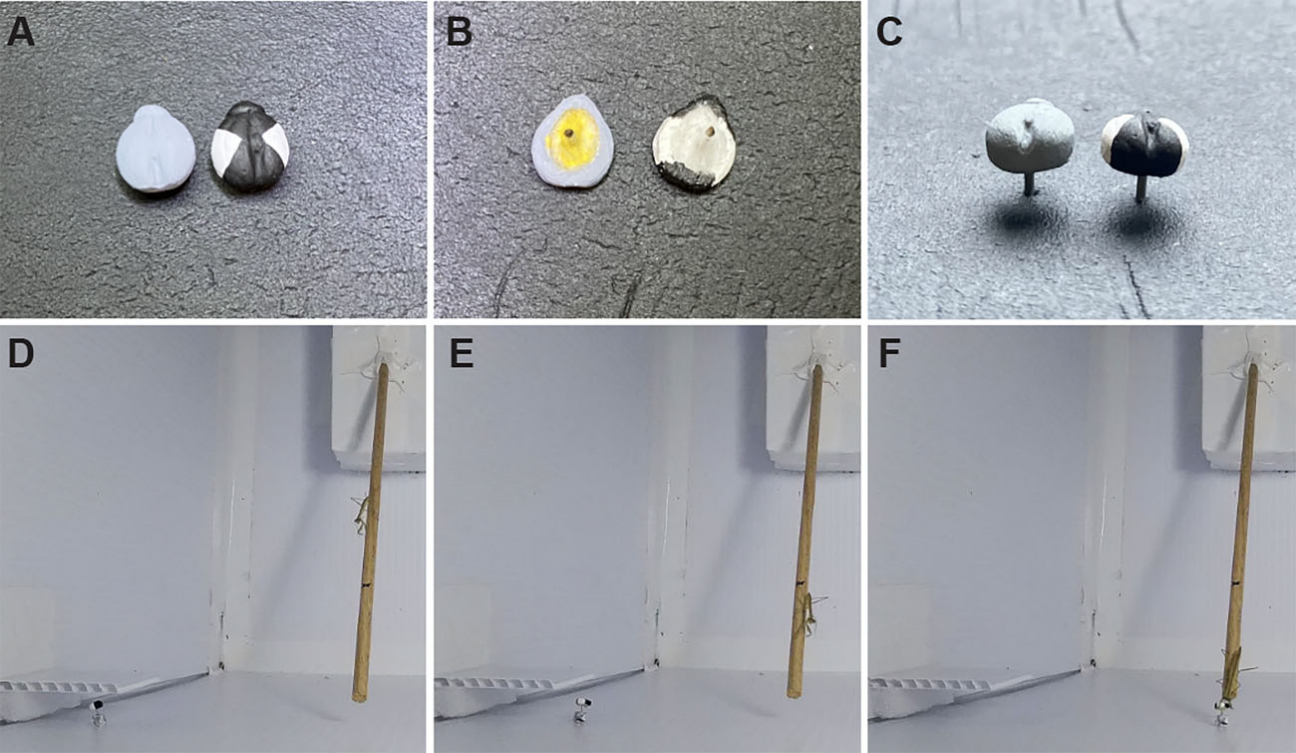
Artificial prey and the experimental paradigm. Images of the artificial prey are shown displaying the control and treatment (contrast) visual conditions (**A**), the control and treatment (yellow hemolymph) olfactory conditions (**B**), and the wire posts allowing the presentation of prey (**C**). A three-step time course of a juvenile mantid completing a trial is also shown (**D–F**).

For the visual signal, “Ebony Black” and “Titanium White” acrylic paints (DecoArt, Stanford, KY, USA) were applied to the prey to render a large black-and-white X-shape on the dorsal surface (Fig. 1). Because the elytra of convergent lady beetles are red with black spots that vary in size and number, this high-contrast, monochromatic pattern was selected due to (1) the use of similar artificial prey patterns in previous studies of aposematism (Aronsson and Gamberale-Stille 2009; Halpin et al. 2020), (2) its putative detectability by mantids having monochromatic vision (Prudic et al. 2007; Fabricant and Herberstein 2015), and (3) its reproducibility in hand-painted prey. To ensure that the visual signal tested was the presence or absence of the contrast pattern, and not another visual quality of the prey, the control state was set to a solid gray appearance that had brightness balanced to the prey bearing the visual signal. Brightness was determined by measuring prey reflectance using an FOIS-1 integrating sphere (Ocean Optics, FL, USA) coupled to a bifurcated fiber optic cable (QR400-7-UV-VIS; Ocean Optics, FL, USA). This cable transmitted light from a quartz tungsten halogen white light source (HL-200-FHSA; Ocean Insight, FL, USA) to the prey, returning the reflected light to a spectrometer with ∼0.3-nm optical resolution (R40000CG-UV-NIR; Ocean Insight, FL, USA) operated by Spectrasuite software (Ocean Optics, FL, USA). Brightness was determined by measuring percent reflectance relative to a White Spectralon Disc, a certified reflectance standard (Labsphere, NH, USA) and summing the percent reflectance values of the prey relative to that standard across 400- to 750-nm wavelengths. This wavelength range was relevant to the reflectance spectrum of artificial prey made possible by illumination of the behavioral arena by an LED light ring (see the subsection “Behavioral arena”). To determine the gray shade required to balance brightness between the treatment (visual signal) and control, a gray reflectance standard was created and assessed using methods inspired by Johnsen (2016). Proportions of black paint were mixed with 0%, 25%, 50%, 75%, and 100% white paint, painted onto casted prey, and measured using the rig above. Plotting percent reflectance of the gray standards allowed the required paint proportion to be estimated, indicating that a 95% white-to-black paint mixture would yield the required gray control. This was validated by measuring the control prey percent reflectance, indicating variation of no more than 3% between control and treatment prey, and between replicates of each prey type.

The olfactory signal was provided by the hemolymph of a convergent lady beetle, which has known alkaloid toxicity, as well as distasteful gustatory and olfactory qualities (Wheeler et al. 2015). Lady beetle hemolymph is also yellow in color. Therefore, to conceal the hemolymph but to allow odorant diffusion, collected hemolymph was placed on the backside of the casted prey. Specifically, a single lady beetle was euthanized by decapitation, the expelled hemolymph was collected with a small cotton pad (cut to a diameter smaller than the casted prey), and the pad was placed against the prey's unpainted, ventral surface. The wire post on the prey's ventral surface created distance between the pad and the wall of the behavioral arena, allowing diffusion of the olfactory signal (Fig. 1). To account for any variation in odorant concentration, we had considered creating a master mix of collected fluid as in Rojas et al. (2019), but were unable to achieve this due to coagulation and insolubility of the hemolymph in water. Therefore, we standardized the olfactory signal by defining it as the entire allotment of hemolymph collected from one lady beetle, which was employed within an experimental trial within 5 min of preparation. The control olfactory signal was 5 μL of deionized water applied in the same fashion, to the cotton pad on the backside of the prey.

Finally, four experimental groups of artificial prey were prepared. For a “visual only” group, prey had the contrast pattern and the water odorant. For an “olfactory only” group, prey had the solid gray appearance and the hemolymph odorant. For a “combined” group, prey had the contrast pattern and the hemolymph odorant. For the control group, prey had the solid prey appearance and the water odorant.

### Behavioral arena

**Fig. 2:**
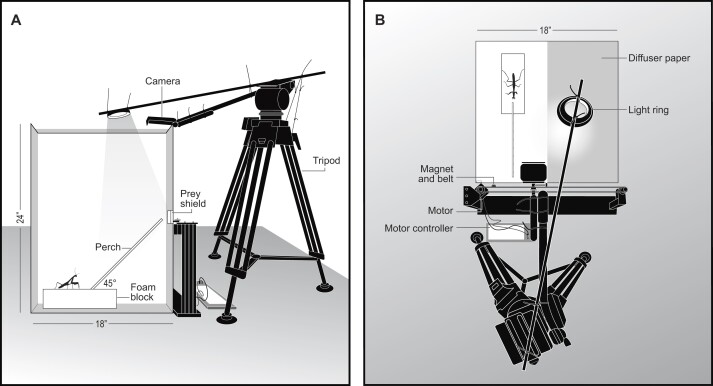
Schematic of the behavioral arena. A side view (**A**) and a top view (**B**) of the arena are shown with all relevant components labeled. Illustration credit: M. D. Smith.

The study was conducted in a closed behavioral room, separate from mantid housing, to avoid disturbances from extraneous auditory or olfactory cues. Experimental trials were run in a white plastic box (46 × 46 × 61 cm, *l* × *w* × *h*) containing a foam block that held a 38-cm-long wooden perch at a ∼45° angle. The perch was marked with a threshold line, roughly two mantid body lengths from the perch end (6 cm for juveniles and 14 cm for adults). The perch terminated 1.5 cm from a viewing wall, which was outfitted with a 5 × 5-cm piece of white plastic for the visual shielding of prey before trials began (Fig. 2). The box was uniformly lit using an LED light ring held behind diffuser paper. Trials were recorded from above by an Insta360 ONE camera (Insta360, CA, USA) set to a frame rate of 60 fps at 2.7K resolution.

Prey were presented on the viewing wall of the arena to appear as crawling lady beetles. This was achieved, first, by pressing the prey's wire post into a piece of foam adhered to a small magnet. The magnet was held up by the force of a larger magnet on the outside of the viewing wall, which was glued to a timing belt (model GT2; Innovaro, Inc., FL, USA) pulled by a stepper motor (model NEMA 17HS4401, Cytron Technologies, Penang, Malaysia). An aluminum sprocket coupled the stepper motor to the belt, which was stretched horizontally along the outside of the viewing wall to another sprocket located on top of a post (Fig. 2). An Arduino motor controller was programmed to move the prey item back and forth over 20 cm of the viewing wall, centered to the perch, at a rate of 3.6 cm/s, which we measured as the rate of a lady beetle crawling.

### Experimental procedure

Juvenile (*n* = 24) and adult (*n* = 23) mantids were randomly assigned in equal sample sizes to the four groups: (1) visual only, (2) odor only, (3) combined visual and odor, and (4) control. As a prior learning event may elicit attack biases to aposematism (Brower 1969; Rowland et al. 2013, 2017), responses of mantids to the assigned groups of artificial prey were examined in a repeated-measures design both before and after being offered live lady beetle prey. The two cohorts of mantids, juveniles and adults, were tested separately at the time they were obtained (see the subsection “Study specimens”), but all mantids experienced the same testing schedule. On Day 1, the mantids were tested in the naïve state. On Day 2, they were offered one convergent lady beetle as prey, and on Day 3, they were trialed again in the experienced state with the same assigned treatment. Lady beetle unpalatability was evidenced by the striking and rapid release of the beetle by both juvenile and adult mantids. We annotated which mantids ultimately consumed the lady beetle versus those that struck, but did not consume the prey. Each day, the order of mantids tested was randomized.

A trial began when a mantid, within its housing container, was placed in the arena for a 3-min acclimation period prior to being placed directly onto the perch. The mantid would begin climbing the perch and when it approached the threshold line, the stepper motor moving the prey would be activated. Two response variables were measured: attack choice (yes/no) and attack latency (s). Attack latency was measured from the time the end of the abdomen crossed the threshold line to the time of the first attack. An attack was defined as an intentional grab, grapple, or strike of the prey item. Animals were given 3 min to attack the prey after crossing the perch before the trial ended and a “no attack” choice was recorded. If a mantid did not cross the threshold line or if it jumped from the perch without orienting to the prey from the perch end, the trial was reattempted. In all cases, the mantids oriented to and tracked the prey in either the first or second attempt. All responses were assessed via two independent reviewers that were blinded to the assigned treatment.

### Data analysis

**Fig. 3:**
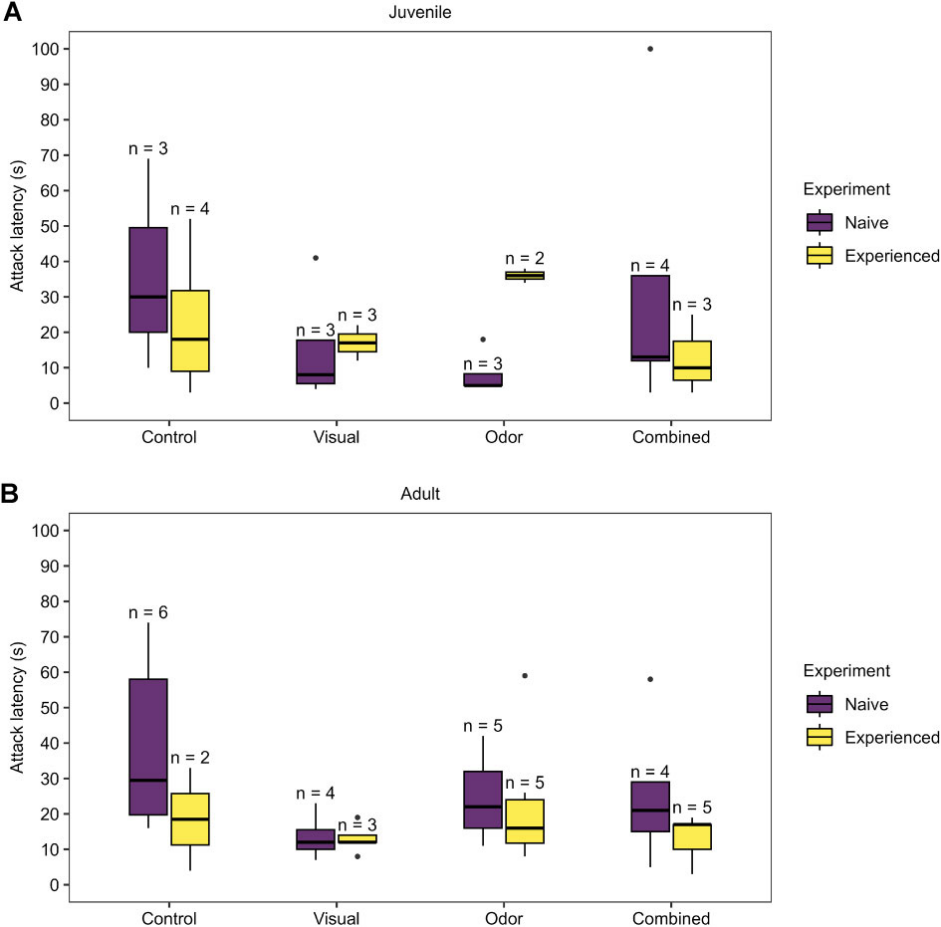
Attack latency of mantids by experience state and developmental stage. Data for juvenile (**A**) and adult (**B**) Carolina mantids show attack latencies to artificial prey across four assigned groups (control, visual signal only, odor signal only, and combined signals) and before and after experiencing live lady beetle prey. Boxes show the median and the 25th and 75th percentiles of data distribution. Vertical lines extend to 1.5 times the interquartile range and black circles indicate outlying values. Note, data are shown for individuals with a positive response of attacking prey, which had to occur within the 3-min trial period.

The data were analyzed in R Statistical Software (v4.1.3; R Core Team 2022) using the “lme4” package (Bates et al. 2015) to fit multiple subtypes of linear models to the data. To determine whether we could combine the data across the entire sample size, we tested whether attack behavior (latency and likelihood) differed between the juvenile and adult cohorts (Table 1: Models 1 and 2). As adults showed a shorter latency and higher likelihood of attack than the juveniles (Table 1: Models 1 and 2), we ran separate models for the two cohorts examining attack latency and likelihood in context of the study hypotheses on experience state and multimodal integration (Table 1: Models 3–6). The model findings indicated small effect sizes, which in part, appeared masked by large variation in the control group (Fig. 3). The longest mean attack latencies were observed in response to the control relative to all other groups (Fig. 3 and Table 1). Thus, to further investigate the differences between the unisensory (visual or olfactory) and multimodal (combined) groups, we ran an additional set of models with the control group removed (Table 1: Models 7–10). Then, to account for possible effects of satiation following consumption of the lady beetle, we tested for effects of ladybug consumption on attack latency and attack likelihood for both cohorts (Table 1: Models 11 and 12), for which none were found. We followed the precedent of using likelihood-based metrics (in lieu of *P* values) to analyze model and predictor performance (see discussion in Bates et al. 2015), and interpreted model performance based on *R*^2^ values and the maximum-likelihood generated 95% confidence intervals (CIs) of predictor variables, with significant effects identified by non-zero CIs. We used binomial logistic regression for models with attack likelihood as a response variable and log-normal regression (using a log link function, not log transformed) for models with attack latency as a response variable because attack latency had a log-normal distribution. Notably, the adult cohort had such a high attack likelihood that for Models 6 and 10 (Table 1), the predictor variables produced subgroups with zero variance.

**Table 1:** Outcomes of the statistical models.^a^

**Model description**		***R* ^2^ metric**	***R* ^2^ value**	**Predictor**	**Predictor mean**	**(Predictor 95% CI)**
**Attack behavior between cohorts**						
1	Attack latency ∼ cohort	*R* ^2^	0.00	Juveniles	25.76	(15.87, 41.81)
				Adults	1.14	(0.63, 2.10)
2	Attacked ∼ cohort	Tjur's *R*^2^	0.09	Juveniles	0.42	(−0.14, 1.02)
				Adults	**1.47**	**(0.49, 2.58)**
**Hypothesis testing with control**						
3	Juvenile attack latency ∼ group + experience	*R* ^2^	0.06	Control and before	37.05	(15.93, 86.15)
				Visual	0.98	(0.35, 2.77)
				Odor	0.43	(0.06, 2.92)
				Combined	0.83	(0.27, 2.56)
				After	0.59	(0.21, 1.66)
4	Adult attack latency ∼ group + experience	*R* ^2^	−0.05	Control and before	49.18	(28.37, 85.26)
				Visual	0.42	(0.14, 1.21)
				Odor	0.40	(0.14, 1.14)
				Both	0.32	(0.09, 1.21)
				After	1.29	(0.63, 2.66)
5	Juvenile attacked ∼ group + experience	Tjur's *R*^2^	0.06	Control and before	0.81	(−0.50, 2.25)
				Visual	0.37	(−1.33, 2.13)
				Odor	−0.35	(−2.05, 1.30)
				Combined	0.37	(−1.33, 2.13)
				After	−0.91	(−2.16, 0.28)
6	Adult attacked ∼ group + experience	Tjur's *R*^2^	0.11	Control and before	1.57	(0.0043, 3.66)^b^
				Visual	NA^c^	NA^c^
				Odor	1.33	(−0.93, 4.45)
				Combined	0.53	(−1.50, 2.75)
				After	−0.86	(−2.97, 0.96)
**Hypothesis testing without control**						
7	Juvenile attack latency ∼ group + experience	*R* ^2^	0.06	Visual and before	36.56	(17.09, 78.25)
				Odor	0.43	(0.06, 3.29)
				Combined	0.85	(0.28, 2.60)
				After	0.56	(0.13, 2.40)
8	Adult attack latency ∼ group + experience	*R* ^2^	0.01	Visual and before	34.80	(19.24, 62.93)
				Odor	0.55	(0.23, 1.31)
				Combined	0.83	(0.35, 1.97)
				After	0.72	(0.28, 1.87)
9	Juvenile attacked ∼ group + experience	Tjur's *R*^2^	0.14	Visual and before	1.56	(0.098, 3.34)
				Odor	−0.79	(−2.66, 0.95)
				Combined	0.00	(−1.84, 1.84)
				After	**−1.53**	**(−3.12, −0.10)**
10	Adult attacked ∼ group + experience	Tjur's *R*^2^	0.07	Visual and before	NA^c^	NA^c^
				Odor	NA^c^	NA^c^
				Combined	NA^c^	NA^c^
				After	0.80	(−1.70, 3.97)
**Accounting for consumption**						
11	Attack latency ∼ consumption + cohort	*R* ^2^	0.02	No ate and juveniles	18.33	(2.28, 147.09)
				Yes ate	1.71	(0.20, 14.82)
				Adults	1.02	(0.50, 2.10)
12	Attacked ∼ consumption + cohort	Tjur's *R*^2^	0.12	No ate and juveniles	0.29	(−1.22, 1.91)
				Yes ate	−0.41	(−2.27, 1.37)
				Adults	**1.67**	**(0.29, 3.21)**

a Linear modeling approaches were used to examine differences between the juvenile and adult cohorts of Carolina mantids and to test hypotheses about the effects of signal groups, experience state, and lady beetle consumption on their attack behavior. Bolded predictor values indicate a significant difference relative to the reference level for that model. CI = confidence interval. NA = not available.

b Outcome due to model non-zero variance.

c Predictor variables with non-zero variance.

## Results

**Fig. 4:**
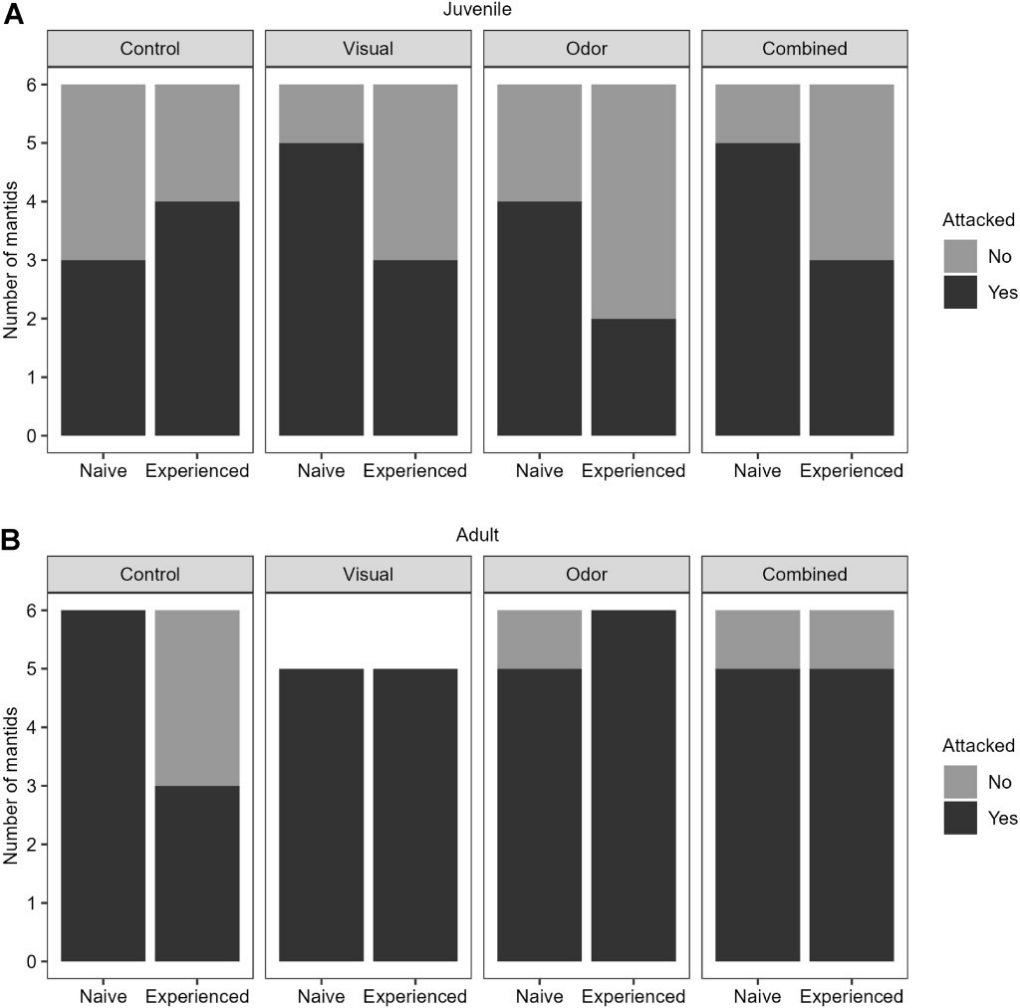
Attack likelihood of mantids by experience state and developmental stage. Data for juvenile (**A**) and adult (**B**) Carolina mantids show attack latencies to artificial prey across four assigned groups (control, visual signal only, odor signal only, and combined signals) and before and after experiencing live lady beetle prey. One animal expired in the adult visual group, resulting in a sample size of *n* = 5, relative to *n* = 6 represented in all other groups.

Prior to testing the study hypotheses, data across trials were combined and compared to test for differences in attack behavior between the two cohorts. For the juvenile mantids, attacks occurred in over 29 of the 48 trials (60%) with a mean of the log-normal distribution of attack latencies of 15.2 s (95% CI [10.3, 22.3]), whereas adults attacked in 40 of the 46 trials (87%) with a mean of the log-normal distribution of attack latencies of 19.2 s (95% CI [14.7, 25.2]) (Figs. 3 and 4). Models comparing the cohorts indicated no difference in attack latency (Table 1: Model 1), but did show a difference in attack likelihood (Table 1: Model 2), with an overall lower attack likelihood of juveniles compared to the adults (Fig. 4 and Table 1).

To address our hypotheses, we then tested the effects of experience state and signal group on attack behavior separately for each cohort. For both cohorts, there were no significant differences in attack latency (Table 1: Models 3 and 4) or in attack likelihood (Table 1: Models 5 and 6) between the experience states or between signal groups. These findings suggested small effect sizes of the aposematic signals that appeared masked by the large latencies and variation in response to the control condition (Figs. 3 and 4 and Table 1). Again, the longest mean attack latencies were observed in response to the control relative to all other groups (Fig. 3), with no significant differences in attack behavior between the control and signal groups for either cohort (Table 1).

We thus removed the control group to increase power in testing the effects of the experimental groups (visual, olfactory, and combined), which revealed a significant change in attack behavior that was dependent on cohort (i.e., developmental stage) and experience state. Specifically, no differences were detected in attack latency of the aposematic (i.e., non-control) prey for both cohorts (Table 1: Models 7 and 8), nor for the attack likelihood of aposematic prey by adults (Table 1: Model 10). For juveniles however, mantids became overall less likely to attack the aposematic prey after the learning event in which they had experienced live lady beetle prey (Table 1: Model 9). For naïve juveniles in treatment groups, 14 (78%) attacked with a mean of the log-normal distribution of attack latencies of 15.0 s (95% CI [8.6, 26.2]). After the learning event, however, only 8 (44%) attacked with a mean of the log-normal distribution of attack latencies of 15.5 s (95% CI [9.2, 26.0]). By contrast, 21 adults (91%) attacked in the naïve state and 19 (83%) attacked in the experienced state, with their mean of the log-normal distribution of attack latencies between the two states being 23.3 s (95% CI [16.5, 32.8]) and 15.6 s (95% CI [10.3, 23.6]), respectively. Finally, the change in attack likelihood detected for juveniles was not explained by satiation. Whereas all 23 adults (100%) consumed the live lady beetle prey, only 17 juveniles (71%) did so, with the remaining 7 juveniles striking, but not consuming, the prey. The models testing the effects of lady beetle consumption on attack behavior only indicated that adults attacked more often (Table 1: Model 12), but showed no significant changes to either attack latency or attack likelihood after consumption by either cohort (Table 1: Models 11 and 12).

Finally, examining for differences in attack latency or likelihood between signal groups, no differences were detected in attack latency (Table 1: Models 7 and 8), nor in attack likelihood (Table 1: Models 9 and 10) between the groups for either cohort. Thus, the attack outcomes between all signal groups were indistinguishable, with no differences in attack behavior of mantids to the presentation of unisensory versus multimodal signals or between either unisensory signal presented alone.

## Discussion

We examined the attack behavior of a mantid predator to aposematic signals to test whether responses are altered by prior experience with aposematism and whether mantids employ multimodal signal integration of combined signals. It appears that mantids respond to aposematic signals and employ attack biases against these signals, but only after prior exposure to aposematism (learning) and only as juveniles. While support is lacking for multimodal integration, this study is the first to suggest a response of mantids to an olfactory warning signal (in addition to a visual warning signal) and to suggest a developmental window for mantid predators to develop biases against aposematism that are shaped by experience.

### Mantid attack behavior and responses to the control

Comparison of the two cohorts indicated that juveniles had an overall lower attack likelihood relative to adults. While studies of ontogenetic changes in mantid attack behavior are limited, at least one study of *Orthodera novaezealandiae* indicated a positive correlation between attack rate and body weight, as well as a marginal increase in attack likelihood between subadult and adult individuals of this species (Fisher et al. 2020). Though the relevance of these findings may be species and sex dependent (Fisher et al. 2020), this study provides context for understanding the higher attack rate observed in adults—perhaps relating to higher caloric requirements, lower risk of predation, or increased aggression relating to sexual cannibalism as seen in other studies of arthropod predation (Arnqvist and Henriksson 1997; Johnson and Sih 2005; Fisher et al. 2020). Due to this finding, we tested the data of the two cohorts separately.

Initial models testing the effects of experience states and signal groups on mantid attack behavior revealed no significant differences in attack latency or likelihood for either cohort. Examination of the data indicated that the variation in attack latency was dominated by responses to the control stimulus, showing the greatest spread and absolute highest latencies of all responses obtained across the signal groups. The reason for this attack ambivalence is unknown, except that control conditions may not have elicited natural attack behavior. In similar studies, a green color of artificial prey has been used as a cue of palatability (e.g., Vickers and Taylor 2018, 2020), as opposed to the solid gray color used here due to the putative monochromatic vision of this species. Though balanced in brightness (to the contrast pattern), this control may not have appeared palatable, but rather, featureless. Thus, due to the inefficacy of this stimulus and to unmask any outcomes across the signal groups, we removed the control dataset for subsequent analysis.

### Prior experience and multimodal integration

Examining the data of the signal groups, our first hypothesis was supported, finding that prior experience with aposematic signal altered attack responses of mantids to the aposematic prey. Specifically, juvenile mantids were less likely to attack the aposematic prey after prior experience with lady beetle aposematism. In these experiences, all mantids struck the live lady beetles, with 17 of 24 (71%) fully consuming the prey. Thus, all animals were exposed to the lady beetle's chemical defense, which may have activated a latent bias (Rowe and Guilford 1999a; Skelhorn et al. 2008) or facilitated avoidance learning (Rowe and Skelhorn 2005; Skelhorn et al. 2016; Stuckert and Summers, 2023) of lady beetle aposematism. Either of these prospects would be supported by the literature, with previous studies of avian predators showing reduced willingness to attack conspicuous (or aposematic) prey after experiencing the prey's chemical defense through predation (Brower 1969; Rowland et al. 2013, 2017). In one study of aversive learning in mantids (*Tenodera aridifolia*; Carle et al. 2018), similar changes in attack behavior were observed before and after such a learning event. In a naïve state, mantids attacked conspicuous prey (bees) at a greater rate than non-conspicuous mealworms. After injection of prey with a bitter compound however, attack rates for both prey items reduced, with consumption of bees, but not mealworms, ceasing entirely. These results align with our findings of decreased attack likelihood of aposematic prey by mantids after experiencing aposematism.

Initial willingness to attack conspicuous prey, despite bearing classical aposematic signals, has been suggested to reduce the efficacy of Batesian mimics (Aubier and Sheratt 2020), which deceptively advertise defense to capitalize on reduced attack rates of aposematic prey (Mappes and Alatalo 1997). Attack willingness in naïve animals increases the likelihood of capturing a suitable meal, while hesitancy after an adverse experience reduces the likelihood of the next deleterious encounter. Predator memory of avoidance learning, or how long the attack bias is retained after experiencing defense, is context specific (e.g., Johnston and Burne 2008; Kang et al. 2016; Ko et al. 2020) and requires further study for avian and arthropod predators, alike.

Unlike our first hypothesis, our second hypothesis was not supported, with comparisons of attack behavior data between signal groups showing no evidence of multimodal integration. Specifically, combined signals did not elicit attack latencies or likelihoods that differed from either unisensory signal presented alone. Specifically, multimodal integration was not detected before or after experiencing lady beetle aposematism nor when comparing the signal groups with or without the control. It is possible that mantids do not employ integration when assessing aposematic signals or alternatively, that our effect sizes were too small to elucidate a synthesized response. As sit-and-wait predators, mantids may strike any prey that becomes available regardless of wariness (Carle et al. 2018) and thus, consumption of edible prey as the dependent variable (as opposed to attack latency of artificial prey) or repeated presentations of prey (as in Berenbaum and Miliczky 1984; Bowdish and Bultman 1993; Carle et al. 2018) during future experiments may reveal greater effects of multimodal integration. Increasing sample size would also increase statistical power, and in attempting to do so here, we obtained unexpected outcomes of this work through the incorporation of the second cohort at an adult life-history stage.

Though not an *a priori* hypothesis, the results of the study indicated differences in attack behavior to aposematic signals with developmental stages between cohorts. Specifically, experience with live convergent lady beetle prey elicited an attack bias in juvenile mantids but not adults. The implication of this finding warrants further investigation because it is unclear whether temporal differences in the acquisition of the two cohorts contributed to this result in addition to any biological differences occurring between the two life-history stages. One possibility, however, is that the stronger experiential effect on juveniles was due to the relatively larger size of the lady beetle prey, such that toxicity was more impactful on the smaller body size of juveniles compared to adults. An alternative explanation, and one provided support by previous studies in the aposematism literature, is that the difference between juveniles and adults is explained by a developmental window of learning or for encoding aposematic wariness that may be tied to changes between life-history stages.

At least in the context of social learning, reduced capacity for avoidance learning of aposematic prey is observed in adults relative to juveniles in domestic chicks (*Gallus gallus*; Johnston et al. 1998; Sherwin et al. 2002), which may be tied to changes in learning mechanisms during ontogeny (Landová et al. 2017). While this has been observed in the avian literature, to our knowledge, it is the first such documentation for an arthropod predator.

## Conclusions

Our finding that attack behavior toward aposematic prey is dependent on experience state and developmental stage indicates overlapping dynamics between arthropod and avian systems in the predator psychology of aposematism. Taken with other studies of arthropod predation (Carle et al. 2018; Vickers and Taylor 2018, 2020), there appear to be unifying principles that underlie aposematic predator–prey interactions independent of taxonomic origin. While future research is needed on the integration of aposematic signals and prior experience in predator psychology (Sherratt 2002), this work adds to our growing knowledge of arthropod attack behavior and suggests mantids as a promising system for studying the co-evolutionary processes of aposematism between predators and prey.

## Data Availability

All relevant data can be found within the article and raw data are available upon request.

## References

[bib1] Arnqvist G , Henriksson S. 1997. Sexual cannibalism in the fishing spider and a model for the evolution of sexual cannibalism based on genetic constraints. Evol Ecol 11:255–73.

[bib2] Aronsson M , Gamberale-Stille G. 2009. Importance of internal pattern contrast and contrast against the background in aposematic signals. Behav Ecol 20:1356–62.

[bib3] Aubier TG , Sherratt TN. 2020. State-dependent decision-making by predators and its consequences for mimicry. Am Nat 196:E127–44. 33064589 10.1086/710568

[bib4] Bates D , Maechler M, Bolker B, Walker S. 2015. Fitting linear mixed-effects models using lme4. J Stat Softw 67:1–48.

[bib5] Berenbaum MR , Miliczky E. 1984. Mantids and milkweed bugs: efficacy of aposematic coloration against invertebrate predators. Am Midl Nat 111:64–8.

[bib6] Bowdish TI , Bultman TL. 1993. Visual cues used by mantids in learning aversion to aposematically colored prey. Am Midl Nat 129:215–22.

[bib7] Brower LP . 1969. Ecological chemistry. Sci Am 220:22–9. 5767170 10.1038/scientificamerican0269-22

[bib8] Carle T , Horiwaki R, Hurlbert A, Yamawaki Y. 2018. Aversive learning in the praying mantis (*Tenodera aridifolia*), a sit and wait predator. J Insect Behav 31:158–75. 29628622 10.1007/s10905-018-9665-1 PMC5882761

[bib9] Carle T , Yamawaki Y, Watanabe H, Yokohari F. 2014. Antennal development in the praying mantis (*Tenodera aridifolia*) highlights multitudinous processes in hemimetabolous insect species. PLoS One 9:e98324. 24896610 10.1371/journal.pone.0098324 PMC4045715

[bib10] Cott HB . 1940. Adaptive coloration in animals. London: Methuen & Co.

[bib11] Duong TM , Gomez AB, Sherratt TN. 2017. Response of adult dragonflies to artificial prey of different size and colour. PLoS One 12:e0179483. 28662042 10.1371/journal.pone.0179483 PMC5491015

[bib12] Edmunds M . 1974. Defence in animals: a survey of anti-predator defences. Harlow: Longman.

[bib13] Ezaki K , Yamashita T, Carle T, Watanabe H, Yokohari F, Yamawaki Y. 2021. Aldehyde-specific responses of olfactory sensory neurons in the praying mantis. Sci Rep 11:1856. 33473161 10.1038/s41598-021-81359-5 PMC7817670

[bib14] Fabricant SA , Herberstein ME. 2015. Hidden in plain orange: aposematic coloration is cryptic to a colorblind insect predator. Behav Ecol 26:38–44.

[bib15] Feller KD , Sharkey CR, McDuffee-Altekruse A, Bracken-Grissom HD, Lord NP, Porter ML, Schweikert LE. 2021. Surf and turf vision: patterns and predictors of visual acuity in compound eye evolution. Arthropod Struct Dev 60:101002. 33191145 10.1016/j.asd.2020.101002

[bib16] Fisher AM , Holwell GI, Price TA. 2020. Behavioural correlations and aggression in praying mantids. Behav Ecol Sociobiol 74:1–10.

[bib17] Gittleman JL , Harvey PH. 1980. Why are distasteful prey not cryptic? Nature 286:149–50.

[bib18] Guilford T . 1990. The evolution of aposematism. In: Evans DL, Schmidt JO, editors. Insect defenses: adaptive mechanisms and strategies of prey and predators. Albany (NY): State University of New York Press. p. 23–61.

[bib19] Guilford T . 1992. Predator psychology and the evolution of prey coloration. In: Crawley MJ, editor. Natural enemies: the population biology of predators, parasites and diseases. Hoboken (NJ): Wiley-Blackwell. p. 375–94.

[bib20] Guilford T , Dawkins MS. 1991. Receiver psychology and the evolution of animal signals. Anim Behav 42:1–14.

[bib21] Halpin CG , Penacchio O, Lovell PG, Cuthill IC, Harris JM, Skelhorn J, Rowe C. 2020. Pattern contrast influences wariness in naïve predators towards aposematic patterns. Sci Rep 10:9246. 32514003 10.1038/s41598-020-65754-y PMC7280217

[bib22] Hebets EA , Papaj DR. 2005. Complex signal function: developing a framework of testable hypotheses. Behav Ecol Sociobiol 57:197–214.

[bib23] Jetz W , Rowe C, Guilford T. 2001. Non-warning odors trigger innate color aversions—as long as they are novel. Behav Ecol 12:134–9.

[bib24] Johnsen S . 2016. How to measure color using spectrometers and calibrated photographs. J Exp Biol 219:772–8. 26985049 10.1242/jeb.124008

[bib25] Johnson JC , Sih A. 2005. Precopulatory sexual cannibalism in fishing spiders (*Dolomedes triton*): a role for behavioral syndromes. Behav Ecol Sociobiol 58:390–6.

[bib26] Johnston AN , Burne TH. 2008. Aposematic colouration enhances memory formation in domestic chicks trained in a weak passive avoidance learning paradigm. Brain Res Bull 76:313–6. 18498948 10.1016/j.brainresbull.2008.02.016

[bib27] Johnston ANB , Burne THJ, Rose SPR. 1998. Observational learning in day-old chicks using one-trial passive avoidance learning paradigm. Anim Behav 56:1347–53. 9933530 10.1006/anbe.1998.0901

[bib28] Kang C , Cho HJ, Lee SI, Jablonski PG. 2016. Post-attack aposematic display in prey facilitates predator avoidance learning. Front Ecol Evol 4:35.

[bib29] Kauppinen J , Mappes J. 2003. Why are wasps so intimidating: field experiments on hunting dragonflies (Odonata: *Aeshna grandis*). Anim Behav 66:505–11.

[bib30] Ko YW , Liao CP, Clark RW, Hsu JY, Tseng HY, Huang WS. 2020. Aposematic coloration of prey enhances memory retention in an agamid lizard. Anim Behav 161:1–13.

[bib31] Land MF . 1997. Visual acuity in insects. Annu Rev Entomol 42:147–77. 15012311 10.1146/annurev.ento.42.1.147

[bib32] Landová E , Hotová Svádová K, Fuchs R, Štys P, Exnerová A. 2017. The effect of social learning on avoidance of aposematic prey in juvenile great tits (*Parus major*). Anim Cogn 20:855–66. 28639012 10.1007/s10071-017-1106-6

[bib33] Lindström L , Rowe C, Guilford T. 2001. Pyrazine odour biases food selection in avian predators against conspicuously coloured prey. P R Soc Lond B Bio 268:357–61.

[bib34] Lutz FE . 1935. Fieldbook of insects. New York (NY): G. P. Putnam's Sons.

[bib35] Mappes J , Alatalo RV. 1997. Batesian mimicry and signal accuracy. Evolution 51:2050–3. 28565127 10.1111/j.1558-5646.1997.tb05129.x

[bib36] Marples NM , Roper TJ. 1996. Effects of novel colour and smell on the response of naive chicks towards food and water. Anim Behav 51:1417–24.

[bib37] Miller CT , Bee MA. 2012. Receiver psychology turns 20: is it time for a broader approach? Anim Behav 83:331–43. 24013277 10.1016/j.anbehav.2011.11.025 PMC3763864

[bib38] Mongeau JM , Schweikert LE, Davis AL, Reichert MS, Kanwal JK. 2021. Multimodal integration across spatiotemporal scales to guide invertebrate locomotion. Integr Comp Biol 61:842–53. 34009312 10.1093/icb/icab041

[bib39] Partan SR . 2013. Ten unanswered questions in multimodal communication. Behav Ecol Sociobiol 67:1523–39. 23956487 10.1007/s00265-013-1565-y PMC3742419

[bib40] Prudic KL , Skemp AK, Papaj DR. 2007. Aposematic coloration, luminance contrast, and the benefits of conspicuousness. Behav Ecol 18:41–6.

[bib41] R Core Team . 2022. R: a language and environment for statistical computing. Vienna: R Foundation for Statistical Computing (https://www.r-project.org/).

[bib42] Rashed A , Beatty CD, Forbes MR, Sherratt TN. 2005. Prey selection by dragonflies in relation to prey size and wasp-like colours and patterns. Anim Behav 70:1195–202.

[bib43] Raška J , Štys P, Exnerová A. 2017. How variation in prey aposematic signals affects avoidance learning, generalization and memory of a salticid spider. Anim Behav 130:107–17.

[bib44] Rojas B , Mappes J, Burdfield-Steel E. 2019. Multiple modalities in insect warning displays have additive effects against wild avian predators. Behav Ecol Sociobiol 73:37.

[bib45] Roper TJ , Cook SE. 1989. Responses of chicks to brightly coloured insect prey. Behaviour 110:276–93.

[bib46] Rossel S . 1979. Regional differences in photoreceptor performance in the eye of the praying mantis. J Comp Physiol 131:95–112.

[bib47] Rowe C . 1999. Receiver psychology and the evolution of multicomponent signals. Anim Behav 58:921–31. 10564594 10.1006/anbe.1999.1242

[bib48] Rowe C , Guilford T. 1996. Hidden colour aversions in domestic chicks triggered by pyrazine odours of insect warning displays. Nature 383:520–2.

[bib49] Rowe C , Guilford T. 1999a. The evolution of multimodal warning displays. Evol Ecol 13:655–71.

[bib50] Rowe C , Guilford T. 1999b. Novelty effects in a multimodal warning signal. Anim Behav 57:341–6. 10049473 10.1006/anbe.1998.0974

[bib51] Rowe C , Halpin C. 2013. Why are warning displays multimodal? Behav Ecol Sociobiol 67:1425–39.

[bib52] Rowe C , Skelhorn J. 2005. Colour biases are a question of taste. Anim Behav 69:587–94.

[bib53] Rowland HM , Fulford AJ, Ruxton GD. 2017. Predator learning differences affect the survival of chemically defended prey. Anim Behav 124:65–74.

[bib54] Rowland HM , Ruxton GD, Skelhorn J. 2013. Bitter taste enhances predatory biases against aggregations of prey with warning coloration. Behav Ecol 24:942–8.

[bib55] Rubi TL , Stephens DW. 2016. Does multimodality per se improve receiver performance? An explicit comparison of multimodal versus unimodal complex signals in a learned signal following task. Behav Ecol Sociobiol 70:409–16.

[bib56] Ruxton GD , Sherratt TN, Speed MP. 2004. Avoiding attack: the evolutionary ecology of crypsis, warning signals and mimicry. Oxford: Oxford University Press.

[bib57] Schuler W , Hesse E. 1985. On the function of warning coloration: a black and yellow pattern inhibits prey-attack by naive domestic chicks. Behav Ecol Sociobiol 16:249–55.

[bib58] Segovia JMG , Pekár S. 2023. Aversive reactions of two invertebrate predators to European red–black insects. Ethology 129:24–32.

[bib59] Sethuraman A , Janzen FJ, Obrycki J. 2015. Population genetics of the predatory lady beetle *Hippodamia convergens*. Biol Control 84:1–10.

[bib60] Sherratt TN . 2002. The coevolution of warning signals. P R Soc Lond B Bio 269:741–6. 10.1098/rspb.2001.1944 PMC1690947 11934367

[bib61] Sherwin CM , Heyes CM, Nicol CJ. 2002. Social learning influences the preferences of domestic hens for novel food. Anim Behav 63:933–42.

[bib62] Skelhorn J , Griksaitis D, Rowe C. 2008. Colour biases are more than a question of taste. Anim Behav 75:827–35.

[bib63] Skelhorn J , Halpin C, Rowe C. 2016. Learning about aposematic prey. Behav Ecol 27:955–64.

[bib64] Skow CD , Jakob EM. 2006. Jumping spiders attend to context during learned avoidance of aposematic prey. Behav Ecol 17:34–40.

[bib65] Sontag C . 1971. Spectral sensitivity studies on the visual system of the praying mantis, *Tenodera sinensis*. J Gen Physiol 57:93–112. 5539340 10.1085/jgp.57.1.93 PMC2203092

[bib66] Stuckert AM , Summers K. 2023. Investigating signal modalities of aposematism in a poison frog. J Evol Biol 36:1003–9. 36309965 10.1111/jeb.14111

[bib67] Vickers ME , Taylor LA. 2018. Odor alters color preference in a foraging jumping spider. Behav Ecol 29:833–9. 30018487 10.1093/beheco/ary068 PMC6041943

[bib68] Vickers ME , Taylor LA. 2020. Hemipteran defensive odors trigger predictable color biases in jumping spider predators. Sci Rep 10:21898. 33318578 10.1038/s41598-020-78952-5 PMC7736339

[bib69] Wheeler CA , Millar JG, Cardé RT. 2015. Multimodal signal interactions in the ladybeetle, *Hippodamia convergens*, aposematic system. Chemoecology 25:123–33.

[bib70] Winters AE , Lommi J, Kirvesoja J, Nokelainen O, Mappes J. 2021. Multimodal aposematic defenses through the predation sequence. Front Ecol Evol 9:657740.

